# Current Conceptual Understanding of the Epileptogenic Network From Stereoelectroencephalography-Based Connectivity Inferences

**DOI:** 10.3389/fneur.2020.569699

**Published:** 2020-11-25

**Authors:** Kanupriya Gupta, Pulkit Grover, Taylor J. Abel

**Affiliations:** ^1^University of Pittsburgh School of Medicine, Pittsburgh, PA, United States; ^2^Department of Neurological Surgery, University of Pittsburgh, Pittsburgh, PA, United States; ^3^Center for the Neural Basis of Cognition, Carnegie Mellon University/University of Pittsburgh, Pittsburgh, PA, United States; ^4^Department of Electrical and Computer Engineering, Carnegie Mellon University, Pittsburgh, PA, United States; ^5^Department of Bioengineering, University of Pittsburgh, Pittsburgh, PA, United States

**Keywords:** focal epilepsy, SEEG, connectivity, epileptogenic, Granger causality, EZ, biomarker

## Abstract

Localization of the epileptogenic zone (EZ) is crucial in the surgical treatment of focal epilepsy. Recently, EEG studies have revealed that the EZ exhibits abnormal connectivity, which has led investigators to now consider connectivity as a biomarker to localize the EZ. Further, abnormal connectivity of the EZ may provide an explanation for the impact of focal epilepsy on more widespread brain networks involved in typical cognition and development. Stereo-electroencephalography (sEEG) is a well-established method for localizing the EZ that has recently been applied to examine altered brain connectivity in epilepsy. In this manuscript, we review recent computational methods for identifying the EZ using sEEG connectivity. Findings from previous sEEG studies indicate that during interictal periods, the EZ is prone to seizure generation but concurrently receives inward connectivity preventing seizures. At seizure onset, this control is lost, allowing seizure activity to spread from the EZ. Regulatory areas within the EZ may be important for subsequently ending the seizure. After the seizure, the EZ appears to regain its influence on the network, which may be how it is able to regenerate epileptiform activity. However, more research is needed on the dynamic connectivity of the EZ in order to build a biomarker for EZ localization. Such a biomarker would allow for patients undergoing sEEG to have electrode implantation, localization of the EZ, and resection in a fraction of the time currently needed, preventing patients from having to endure long hospital stays and induced seizures.

## Introduction

Focal epilepsy is the most common type of epilepsy (1). Although seizure onset is confined to a focus comprising one or a few areas, several lines of evidence now demonstrate that focal epilepsy is a network disorder with widespread influence rather than a disorder of an isolated area (2, 3). The epileptogenic zone (EZ), often defined theoretically as that part of the cortex which when removed results in seizure freedom, must be localized in order to successfully treat patients with drug-resistant focal epilepsy (4). However, such a definition of the EZ cannot be measured, as it is impossible to know whether a smaller area could have been resected that would have also provided seizure freedom for a patient. Here, we operationalize the EZ as the area of seizure onset and primary seizure organization (5). Neuroimaging modalities such as fMRI and EEG have allowed us to examine how the EZ exhibits abnormal connectivity. By using network models to understand focal epilepsy, we can find new ways to define and identify the EZ. In this manuscript, we describe emerging connectivity methods for identifying the EZ measured by sEEG (Table 1) and conceptual advances of the EZ using these methods.

**Table 1:** sEEG functional connectivity methods and their advantages and limitations.

**Type**	**Description**	**Advantages**	**Limitations**
**UNDIRECTED, COHERENCE-BASED CONNECTIVITY METHODS**			
Pearson correlation coefficient	A measure of the linear relationship between time series.	•Simple to implement	•No directionality (cross-correlation, which measures correlation at different time lags, can measure directionality) •Assumes a linear relationship between time series
Phase locking value	A measure which quantifies the phase synchrony between two different signals in a certain frequency band (6), or the cross-frequency coupling in one time series (7–10).	•Can distinguish between the roles of phase and amplitude in a signal	•No directionality
Imaginary coherence	A measure of functional connectivity that includes first calculating spectral coherence, which measures the explained variance in a sEEG signal by another signal within a specific frequency band (11). Only the imaginary component is used for analysis, which discards coherence that occurs at zero phase lag.	•Avoids false connectivity detection due to volume conduction/field spread	•No directionality [a different measure, Phase Slope Index, can measure directionality (12)]
**Type**	**Description**	**Advantages**	**Limitations**
**DIRECTED CONNECTIVITY METHODS**			
Partial directed coherence	A measure of directional connectivity that is based off of the concept of Granger causality. A multivariate autoregressive model is transformed into the frequency domain to perform this analysis (13).	•Each electrode contact's PDC value to another contact is normalized by the sum of the outflow from the contact, which highlights contacts that receive a high degree of inflow.	•Assumes linearity and stationarity of sEEG data •Unclear how Granger causality reflects actual causality
Directed transfer function	A measure of directional connectivity that is based off of Granger causality. A multivariate autoregressive model is transformed into the frequency domain to perform this analysis (14).	•Each value of DTF from electrode contact x → y is normalized by the sum of the inflow to y, which highlights contacts that send out a high degree of outflow.	•Assumes linearity and stationarity of sEEG data •Unclear how Granger causality reflects actual causality
Non-linear correlation coefficient (h^2^)	A measure of the dependence between time series that takes into account both linear and non-linear relationships.	•Can measure both the strength and direction of connections, since h^2^ is asymmetric (${\text{h}}_{\text{XY}}^{2}$ is not equal to ${\text{h}}_{\text{YX}}^{2}$) and can be calculated at different time lags (15) •Does not assume a linear relationship between signals	•Heavy computation can be needed (16) •Previously documented small impact of interictal spikes on analysis (17)
Phase transfer entropy	A model-free measure of directed connectivity using phase information (18).	•Can measure directionality •Does not assume a linear relationships between signals •Resistant to spurious connectivity detections due to noise	•A relatively newer measure

Resting-state EEG connectivity studies have also generated new knowledge about the outward connectivity of the EZ. Whereas, the bilateral posterior cingulate cortex (PCC) has been shown to have the highest outward connectivity in controls during resting state, the ipsilateral hippocampus had the highest outward connectivity in patients with temporal lobe epilepsy (TLE) using electrical source imaging from high-density EEG (19). This finding indicates that the hippocampus (the EZ) seems to be the predominant source of influence outflow in TLE even in the absence of seizures, and it may be disrupting other functional networks, such as those controlled by the PCC. In the immediate period leading up to an interictal discharge, patients with right and left TLE show rapidly increasing outward connectivity from the ipsilateral mesial temporal pole, suggesting that epileptogenic areas recruit other brain regions to generate and spread epileptogenic activity (20).

Localization of the EZ is crucial for patients with drug-resistant focal epilepsy who are candidates for surgery. Stereoelectroencephalography (sEEG), a minimally invasive technique used to record electrical activity directly from the brain, is used frequently in patients with drug-resistant epilepsy in which the EZ cannot be localized using non-invasive modalities. Since sEEG exhibits the fine temporal resolution of intracerebral recordings, it provides an unparalleled opportunity to explore the interictal and ictal network properties of the EZ and may provide opportunities to exploit the network properties of the EZ for localization purposes. Furthermore, if successful algorithms were found using only a short period of interictal data, recording time could be reduced from days to only hours at most, and we would not have to trigger seizures in patients. These improvements would be invaluable for patients with epilepsy, especially children who might not tolerate multiple days in the epilepsy monitoring unit.

## sEEG Connectivity Studies

While several studies have examined interictal connectivity using resting-state fMRI (rs-fMRI) and scalp EEG, relatively fewer studies have examined interictal connectivity using sEEG. sEEG offers exquisite spatial and temporal resolution compared to rs-fMRI and scalp EEG, making it well-suited to examine the fine time course of epileptic cortical activity. Unlike scalp EEG, sEEG can measure activity directly from both cortical and subcortical areas and is only limited by the placement of electrodes, which is usually only in areas suspected to be involved in seizure onset or propagation. Whereas, fMRI is reliant on the hemodynamic response, which is a delayed measure associated with brain activation, sEEG provides a real-time measure of electrical brain activity. Compared to subdural grids, another type of invasive monitoring technique which can only be placed on the surface of the cortex, sEEG electrodes can be placed into deep structures of the brain. Although sEEG is more invasive than fMRI and scalp EEG, it does not require a craniotomy like subdural grids and has a good safety profile with a lower rate of postoperative complications and infections compared to grids (21–25). Given that no other technique has the combined high spatiotemporal resolution and safety associated with sEEG, it is an ideal tool for studying epileptogenic networks. Building on a strong premise of previous work, we present connectivity findings from several sEEG papers and provide a framework for a model of ictogenesis that could be used to identify a biomarker for the EZ (26, 27).

## Functional Connectivity Methods and Findings

### Coherence-Related Undirected Methods

Several methods have been used to analyze the functional connectivity between epileptogenic and non-epileptogenic areas (Table 1) during resting-state, pre-ictal, and ictal periods. A recent sEEG study of focal epilepsy measured the Pearson correlation coefficient during resting state and calculated node strength, which measures the average connection between a contact to all other contacts, for each contact (28). The distinguishability between node strength in resected vs. not resected contacts (D_RS_) was calculated for each patient. Only after correcting for spatial proximity between contacts did the D_RS_ values between seizure-free and not-seizure-free patient groups become significantly different. Patients with seizure-free outcomes had less distinguishability than patients who were not seizure-free, suggesting that resected nodes were more similar to unresected nodes and less epileptogenic in seizure-free patients. This finding also suggests that network properties during resting-state can help differentiate patients who will have better outcomes postoperatively.

In another study, alpha, theta, and delta imaginary coherence within a region and between regions was significantly higher in epileptogenic regions compared to non-epileptogenic regions, with the alpha-frequency band showing the biggest differences (29). Furthermore, several graph theory measures, including nodal betweenness centrality, edge betweenness centrality, and clustering coefficient were significantly higher in epileptogenic regions. The increased edge and nodal betweenness centrality of the EZ indicates that the epileptogenic zone is an important hub for network connectivity. The increased clustering coefficient reflects that the EZ and the nodes that it is connected to occur in a cluster, or segregated group, that are all functionally connected to each other. When all of these network measures were combined into a logistic regression model, the model was able to classify epileptogenic regions with an AUC of 0.78. This study suggests that epileptogenic cortex is strongly connected to areas likely involved in primary seizure organization and propagation. Understanding these features of epileptogenic cortex may be exploited to provide a clinically relevant biomarker of the EZ.

Phase-amplitude coupling occurs (PAC) is a connectivity measure based on how the phase of a low-frequency rhythm couples with the amplitude of another high-frequency rhythm. One study used PAC to characterize areas belonging to the ictal core vs. the surrounding penumbra (7). The ictal core was defined as the region of neurons showing hypersynchronous firing in multielectrode array recordings during seizures, whereas the penumbra was defined as a group of areas that show less prominent, unorganized firing. PAC between a low-frequency ictal rhythm and high-gamma frequency amplitude in subdural electrodes was shown to correspond with multiunit firing bursts measured by nearby microelectrode arrays in the ictal core after the onset of seizure activity. However, subdural electrode contacts with low-frequency activity that was not phase-locked to high-gamma amplitude coincided with nearby microelectrode arrays that did not display synchronized firing bursts, suggesting that these contacts are part of the penumbra in which there is a lack of early seizure propagation. The lack of high-frequency oscillations in the penumbra has been thought to be a result of a feedforward inhibitory mechanism, resulting in the penumbra displaying high-amplitude, low-frequency EEG signals without true increases in synchronized neuronal firing (30). Therefore, phase-amplitude coupling between high-frequency oscillations and low-frequency ictal activity appears to be a biomarker for the epileptogenic zone, which can be targeted for resection while sparing the ictal penumbra. Another study used Phase Locking Value (PLV) to identify the seizure onset zone using ictal data in 10 patients with focal epilepsy (8). In seizure-free patients, resected electrodes displayed greater PLV right before seizure onset, and higher PLV peak and power just after seizure onset. This finding further suggests that PAC between low and high-frequency rhythms could be a reflection of the mechanism leading to seizure generation and propagation. Several measures calculated from the PLV were combined into a logistic regression model to classify electrode contacts as belonging to either the seizure onset zone (SOZ) or non-SOZ. Ninety-six percent of the electrodes classified as belonging to the SOZ were within the resection area in seizure-free patients. In patients that were not seizure-free, the more electrodes that were labeled by the algorithm as in the SOZ that were not resected, the worse the patient's seizure outcome.

### Directional Connectivity Methods

Granger causality is a concept that has been used to develop several connectivity measures and is a favorable connectivity analysis method given that it can provide directional inferences of influence. In a study using generalized Partial Directed Coherence (PDC), a Granger causality-based method, on resting-state sEEG data, the region of the highest inflow colocalized with the EZ in nine patients with temporal lobe epilepsy (31). In-degree, a graph theory measure of the number of inward connections to a node, was similarly found to be effective at localizing the EZ in another study when applied to PDC analyses from pre-ictal sEEG data (5 s before seizure onset), as epileptogenic regions had the highest in-degree (32). Together, these studies indicate that there is possibly a control mechanism preventing seizures during seizure-free periods and that during a seizure such influence may be lost. Another study using non-linear regression, an alternative to Granger causality-based methods for studying directed network connectivity, found that in pre-ictal sEEG data (8 s before seizure onset), the total number of connections was found to be better at localizing the epileptogenic zone than the number of inward or outward connections, suggesting that the pre-ictal period could be a transition point between the inward influence received during resting-state and the outward connectivity demonstrated during seizure onset (33). However, it is also possible that inward connectivity toward the EZ drives the EZ to send outward ictogenic connectivity. Epileptogenic structures in patients with mesial TLE also show increased synchronization in the pre-ictal period, which may be a seizure generation mechanism (34).

Contrary to the inward influence received during interictal and pre-ictal periods, the EZ shows a switch in connectivity patterns during seizure onset. At the start of a seizure, the EZ can be identified as the region containing the contact with the highest out-degree (35) or the most causal outflow (36). Using Directed Transfer Function (DTF) connectivity, nodes with high betweenness centrality, a graph theory marker for hubs in a network, during seizures have also been shown to coincide with the seizure onset zone (37). This finding was modulated by frequency, as overlap with the SOZ was greater for nodes with large betweenness centrality values in higher frequency gamma and beta networks compared to theta and alpha networks. The high betweenness centrality of nodes thought to be involved in seizure activity was shown to decrease during a seizure and reach a minimum 1 min after a seizure, perhaps because Granger causal influence is spreading more distally from the EZ as the seizure progresses and cortical activity is synchronizing. However, after a seizure, the betweenness centrality rose to pre-ictal levels in just 5 min after a seizure, emphasizing the need to understand the dynamics of EZ connectivity.

In patients who were seizure-free postsurgery, the SOZ colocalized more with nodes with high betweenness centrality in the gamma band during seizures compared to those of patients who still had seizures after surgery (37). This finding suggests that regions involved in generating epileptogenic activity display high gamma band connectivity in ictal periods, which is supported by phase-amplitude coupling studies mentioned earlier and studies showing that fast gamma band activity and suppression of lower frequency activity during seizure onset can localize the EZ (38, 39). However, the relative contribution of connectivity in higher frequencies during ictal periods, with that in alpha band during resting-state, to the epileptogenicity of the EZ remains to be investigated. Furthermore, patients who did not have seizure freedom after surgery have been shown to have a greater percentage of nodes with high betweenness centrality preoperatively, highlighting that as more regions in the brain are highly connected, and likely to spread epileptogenicity, the less likely the patient will be seizure-free (40). However, if the resected area has nodes with high betweenness centrality during the middle to after the end of the seizure, the patient is less likely to be seizure-free. Hence, within or near the EZ, there may be nodes that should be spared from resection, as they are likely involved in suppressing seizures. Another sEEG study using phase transfer entropy found that the ratio of out/in connections increased from the interictal to ictal state but declined drastically from the late-ictal to post-ictal state, suggesting a need to study the possible role of inward connectivity in seizure control (41).

In a sEEG study using non-linear regression, brain regions were classified as belonging to the EZ, propagation zone (PZ), or non-involved zone (NIZ) using ictal data (42). Then, resting-state connectivity was measured between zones and within a zone itself. This analysis revealed that resting-state connectivity was greater within the EZ compared to the NIZ, and greater within the PZ vs. the NIZ, illustrating that the more epileptogenic the area, the more that area is connected within itself, which could be a mechanism for seizure generation. Furthermore, the connectivity between EZ-PZ was higher than the connectivity between EZ-NIZ, and the connectivity within the EZ was significantly higher than connectivity between the EZ-NIZ but not higher than connectivity between the EZ-PZ. Therefore, the EZ seems to be highly connected to itself and the PZ, but not the NIZ. Whereas, high internal connectivity in the EZ could allow for generation of epileptogenic activity, enhanced EZ-PZ connectivity could allow for quick propagation of epileptogenic activity during a seizure. The EZ was also shown to be the source of outward connectivity toward the PZ and NIZ. Although this seems contradictory to an earlier finding that inflow was able to localize the EZ during resting state, it is possible that the EZ could be receiving inward connectivity while also producing outward connectivity toward propagation zones.

### Cortico-Cortico-Evoked Potentials

Another method that uses directional connectivity to localize the EZ is based on cortico-cortico-evoked potentials (CCEPS). In patients with cortical electrodes, stimulation is applied at one electrode and the resulting evoked potential is measured at other electrode sites. One study illustrated that the amplitude of local CCEPs after stimulation in the SOZ is greater than the amplitude of local CCEPs after stimulation in a region near the SOZ that is not active at seizure onset, suggesting that the epileptogenic cortex is more responsive to excitation due to increased connectivity compared to non-epileptogenic areas (43). A recent study of patients with drug-resistant epilepsy used CCEPs to study directional connectivity between different areas of the brain (44). A large amplitude response evoked from stimulation was taken as a marker for the presence of a directed connection from the stimulation site to the recording site. After constructing connectivity matrices, the authors used graph theory measures to compare the connectivity of the EZ vs. non-EZ. When stimulation was applied to a contact located in the EZ, other electrode contacts in the EZ had a large amplitude evoked potential, reiterating that areas within the EZ are highly connected. Furthermore, the EZ was shown to have higher degree centrality, illustrating that it is highly connected to other nodes in the network (45). Another CCEP paper on patients with focal epilepsy found that contacts in the SOZ had increased out-degree, indicating a higher number of outward connections, as well as increased structural and functional connectivity toward areas of seizure propagation (46).

## Future Directions

There are several promising topics for future investigation. sEEG studies showing the dynamic nature of directional connectivity of the EZ between interictal, pre-ictal, and ictal time periods are needed to demonstrate how the EZ may be prevented from producing seizures during interictal periods and subsequently becomes uncontrollable just before seizure onset. The dynamic connectivity of the EZ could be used to build an individualized model of epileptogenicity for each patient that identifies the number and location of epileptogenic foci, as well as the optimal area of resection that spares regions that may be involved in seizure control.

## Discussion

**Figure 1:**
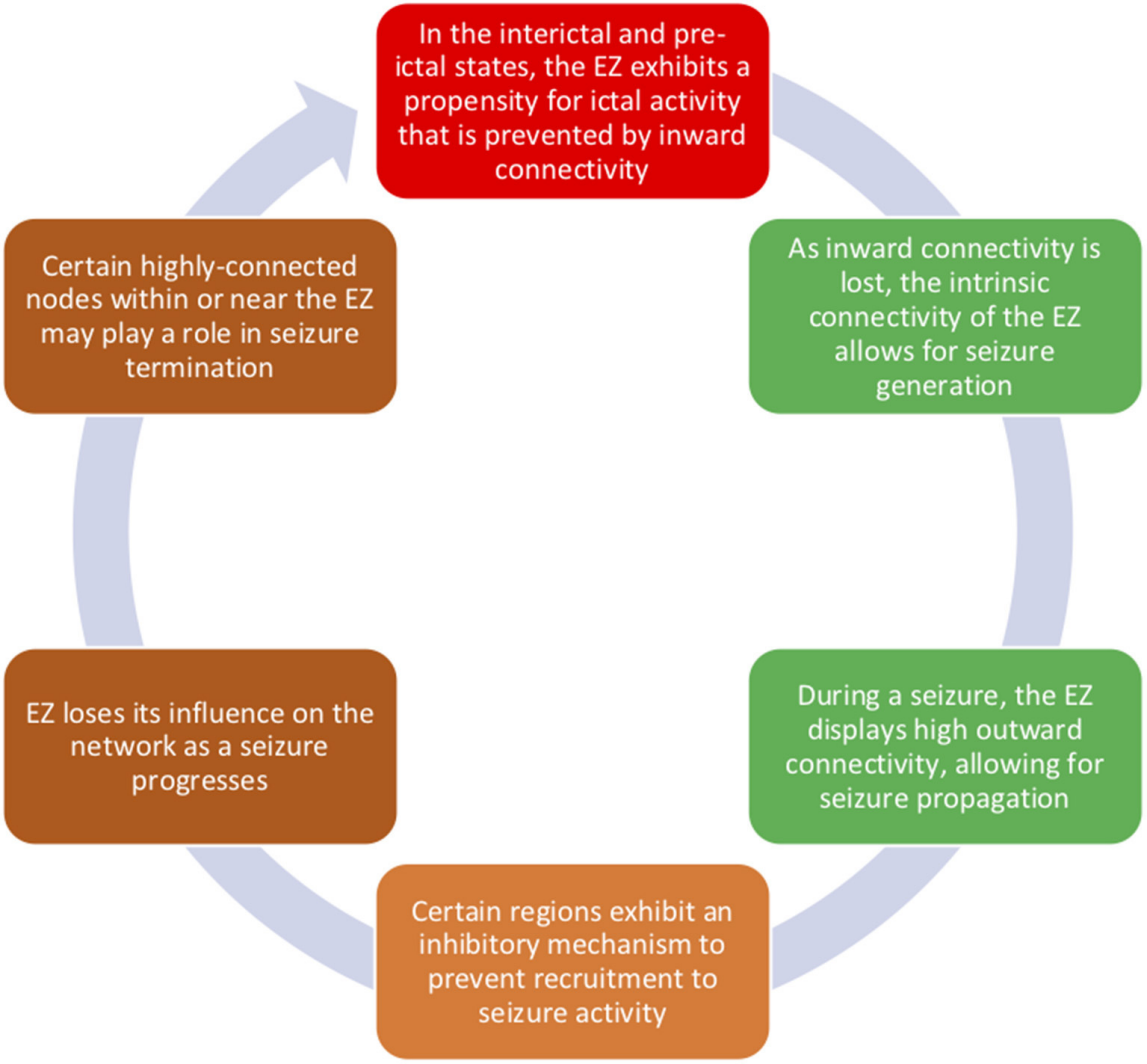
Connectivity of the epileptogenic zone changes between interictal, pre-ictal, and ictal time periods. During interictal periods, the EZ displays both high outward connectivity, which is likely related to the EZ's seizure-generating activity, and inward connectivity, which is possibly preventing a seizure from starting. During seizure onset, we propose that such inward control is lost and that the internal connectivity of the EZ allows for seizure generation. Epileptogenic influence spreads from the EZ via its high connectivity to propagation zones. However, the EZ becomes less influential in the network over time, and regions in or near the EZ may be involved in seizure cessation.

Findings from sEEG studies have allowed us to better understand how focal epilepsy has widespread effects on brain network connectivity. Taken together, sEEG connectivity findings have shed light on a model of ictogenesis (Figure 1): (1) during interictal and pre-ictal periods, the EZ, which may be sending outward connectivity to generate a seizure, is also controlled by inflow from other regions; (2) during seizure onset, the EZ is no longer controlled by inward connectivity and generates epileptiform activity via its high intrinsic connectivity; (3) the EZ sends epileptic influence to propagation zones; (4) areas that are not invaded by seizure activity may be protected by a feedforward inhibition mechanism; (5) as a seizure progresses, the EZ loses its influence on the network, which then builds up again post-ictally; and (6) seizure termination may be partly a result of internal regulation within or near the EZ.

Barriers to implementation of automated resting-state sEEG analysis in the clinic include several parameters that still need to be explored fully and optimized, such as the effect of type of focal epilepsy on suitability for connectivity-based analyses and the amount of resting-state data that would need to be analyzed to localize the EZ confidently. However, the sEEG studies discussed in this paper showing connectivity-based differences between the EZ and non-EZ have been performed on patients with different types of focal epilepsy, and even a few minutes of data has been shown to be sufficient for these analyses. These results show the promise of implementing sEEG-based localization methods of the EZ into clinical practice. Hence, it is exciting that soon we could have automated, intraoperative localization and removal of the EZ that prevents patients from enduring long hospital stays and multiple seizures and gives them a better chance at long-term seizure freedom.

## Author Contributions

KG wrote the initial and revised drafts of the manuscript. PG and TA are equally responsible for reading and editing of the manuscript. All authors contributed to the article and approved the submitted version.

## Conflict of Interest

TA is a consultant for the Monteris Corporation. PG is on the board of Precision Neuro. The remaining author declares that the research was conducted in the absence of any commercial or financialrelationships that could be construed as a potential conflict of interest. The handling Editor declared a shared affiliation, though no other collaboration, with several of the authors KG and TA.
